# Products Released from Structurally Different Dextrans by Bacterial and Fungal Dextranases

**DOI:** 10.3390/foods10020244

**Published:** 2021-01-26

**Authors:** Silke L. Pittrof, Larissa Kaufhold, Anja Fischer, Daniel Wefers

**Affiliations:** 1 Department of Food Chemistry and Phytochemistry, Karlsruhe Institute of Technology, 76131 Karlsruhe, Germany; pittrof.silke@web.de (S.L.P.); issi.kaufhold@gmail.com (L.K.); anja-93-fischer@web.de (A.F.); 2 Food Chemistry–Functional Food, Institute of Chemistry, Martin-Luther-University Halle-Wittenberg, 06120 Halle (Saale), Germany

**Keywords:** glucans, structure, HPAEC-PAD, enzymatic fingerprinting, oligosaccharides, chromatography, enzymes, exopolysaccharides, enzymatic cleavage

## Abstract

Dextran hydrolysis by dextranases is applied in the sugar industry and the medical sector, but it also has a high potential for use in structural analysis of dextrans. However, dextranases are produced by several organisms and thus differ in their properties. The aim of this study was to comparatively investigate the product patterns obtained from the incubation of linear as well as *O*3- and *O*4-branched dextrans with different dextranases. For this purpose, genes encoding for dextranases from *Bacteroides thetaiotaomicron* and *Streptococcus salivarius* were cloned and heterologously expressed in *Escherichia coli*. The two recombinant enzymes as well as two commercial dextranases from *Chaetomium* sp. and *Penicillium* sp. were subsequently used to hydrolyze structurally different dextrans. The hydrolysis products were investigated in detail by HPAEC-PAD. For dextranases from *Chaetomium* sp., *Penicillium* sp., and *Bacteroides thetaiotaomicron*, isomaltose was the end product of the hydrolysis from linear dextrans, whereas *Penicillium* sp. dextranase led to isomaltose and isomaltotetraose. In addition, the latter enzyme also catalyzed a disproportionation reaction when incubated with isomaltotriose. For *O*3- and *O*4-branched dextrans, the fungal dextranases yielded significantly different oligosaccharide patterns than the bacterial enzymes. Overall, the product patterns can be adjusted by choosing the correct enzyme as well as a defined enzyme activity.

## 1. Introduction

Dextrans are versatile polysaccharides that have a high application potential in foods, cosmetics, or medical products [1]. They are formed by dextransucrases which are produced by several lactic acid bacteria. These enzymes are able to hydrolyze sucrose, release fructose, and transfer the intermediately bound glucose units to a growing polysaccharide [2]. Dextrans are characterized by a homogenic backbone of α-1,6-linked glucose units. Depending on the dextransucrase, this backbone may be linear or decorated with mono-, di-, or oligomeric side chains at position *O*2, *O*3, or *O*4, which results in a considerable structural complexity [3,4].

A specific hydrolysis of dextrans can be achieved by the application of endo-dextranases, which hydrolyze the linear, α-1,6-linked areas of the dextran backbone. This results in the conversion of polymeric dextrans to glucose, isomaltose, linear isomalto-oligosaccharides, and, in case of branched dextrans, branched oligosaccharides. The most important application of dextranases is the removal of dextrans during the sugar production process. Furthermore, dextranases have potential for some medical purposes such as the treatment of dental plaque [5]. In addition, endo-dextranase hydrolysis and subsequent analysis of the liberated oligosaccharides can be used for structural analysis of dextrans or mixed-linkage α-glucans [6,7,8,9,10,11,12,13]. Dextranases are expressed by a wide range of microorganisms and have different properties with regards to their optimum incubation conditions as well as their product patterns [5]. However, information on the product patterns of dextranases from varying origins, especially with regards to branched oligosaccharides, is scarce. Therefore, the aim of this study was to comparatively investigate the release of linear and branched oligosaccharides from linear, *O*3-branched, and *O*4-branched dextrans by bacterial and fungal dextranases.

Fungal dextranases are widely applied and commercially available, thus, two commercial fungal dextranases (from *Chaetomium* sp. and *Penicillium* sp.) were used in this study. Dextranases from both species were previously investigated with regards to their optimum incubation conditions and, in part, their hydrolysis products [7,8,14,15,16,17,18,19,20,21,22,23,24]. Because bacterial dextranases may have a significantly different product spectrum, genes encoding for two dextranases were cloned from *Streptococcus (S.) salivarius* DSM 20560 (SSAL_1145) and *Bacteroides (B.) thetaiotaomicron* DSM 2079 (BT_3087). As for the fungal dextranases, some investigations were conducted on the optimum incubation conditions and the hydrolysis products of dextranases from these genera/species [25,26,27,28,29]. For a detailed investigation of the products released by hydrolysis with the bacterial and fungal dextranases, dextrans from *Ligilactobacillus animalis*, *Latilactobacillus curvatus*, and *Limosilactobacillus reuteri* were used (for simplicity, the abbreviation *L*. will be used for all three genera). These dextrans have completely linear, *O*3-branched, and *O*4-branched backbones, respectively [11,30]. Because several oligosaccharides released from these dextrans by *Chaetomium* sp. dextranase were previously isolated and characterized in detail [11,12], an analysis of the hydrolysates by HPAEC-PAD yields detailed information on the product patterns of the dextranases.

## 2. Materials and Methods

### 2.1. General

AZCL-dextran and dextranase from *Chaetomium* sp. (EC 3.2.1.11, 490 U/mg) were purchased from Megazyme (Bray, Ireland). Dextranase from *Penicillium* sp. (EC 3.2.1.11, 100–250 U/mg) was purchased from Sigma-Aldrich (Schnelldorf, Germany). pLIC-SGC1 was a gift from Nicola Burgess-Brown (Addgene plasmid # 39187). If not stated otherwise, all other chemicals used were of “*p*.a.” grade or better and were purchased from Sigma Aldrich, VWR (Radnor, PA, USA), or Carl Roth (Karlsruhe, Germany).

### 2.2. Molecular Cloning and Heterologous Expression

Genes encoding for dextranase from *S. salivarius* DSM 20560 (SSAL_1145; GenBank accession number: AIY20395) and *B. thetaiotaomicron* DSM 2079 (BT_3087; GenBank accession number: AAO78193) were amplified from the respective genomic DNA by using a Phusion High-Fidelity PCR kit (Thermo Fisher Scientific, Waltham, MA, USA). Genomic DNA was purchased from Deutsche Sammlung von Mikroorganismen und Zellkulturen (DSMZ, Braunschweig, Germany) and primers were synthesized by Integrated DNA Technologies (Coralville, IA, USA). For ligation independent cloning of the genes into the pLIC-SGC1 vector, specific overhangs were added at both ends of the genes [31,32,33]. Genes encoding for glucansucrases from *L. animalis* TMW 1.971 and *L. curvatus* TMW 1.624 (previously described by Rühmkorf et al. [34]) were cloned from genomic DNA, while the gene encoding for glucansucrase from *L. reuteri* TMW 1.106 was previously cloned into the pLIC-SGC1 vector [13]. The sequences of the inserted genes were confirmed by Sanger sequencing (Eurofins GATC Biotech, Konstanz, Germany). For protein expression, plasmids were transformed into OneShot BL21 Star (DE3) cells by heat shock. A single colony was transferred to 5 mL of LB medium (100 μg ampicillin/mL) and grown at 37 °C and 225 rpm. After growing the precultures for 6 h at 37 °C and 225 rpm, the suspension was transferred to 200 mL of fresh LB medium (100 μg ampicillin/mL). The culture was further incubated for 3 h at 37 °C and 225 rpm, and isopropyl-β-D-thiogalactopyranoside (final concentration: 0.1 mM) was added to induce protein expression. After incubating for 16 h at room temperature, cells were harvested by centrifugation, re-suspended in binding buffer (50 mM sodium phosphate, 300 mM NaCl, 10 mM imidazole, pH 7.5), and lysed by sonication (amplitude 50%, 3 × 20 s pulse, 59.9 s pause) with a Sonifier W−250 D (Branson, Danbury, CT, USA). Cell debris were removed by centrifugation (30 min, 4 °C, 20,000× *g*) and the clear supernatant was transferred to a HisPur Ni-NTA resin (Thermo Fisher Scientific; pre-equilibrated with binding buffer). The suspension was incubated for 1 h at 4 °C, washed with 4 volumes of binding buffer, and the recombinant proteins were eluted with elution buffer (50 mM sodium phosphate, 300 mM NaCl, 100 mM imidazole, pH 7.5).

### 2.3. Optimum Incubation Conditions

Dextranase activity and optimum incubation conditions were determined by monitoring the hydrolysis of AZCL-dextran according to the manufacturer’s instructions. Briefly, 20 µL of enzyme solution was added to 100 µL of AZCL-dextran suspension in different buffers (pH 4.0–5.5: 50 mM sodium acetate buffer; pH 6.0–7.0: 50 mM sodium phosphate buffer; pH 7.5 and 8.0: 50 mM Tris buffer), which were pre-equilibrated at the desired temperature. Subsequently, the suspension was incubated for 15 min (temperature from 20–90 °C). The reaction was stopped by adding 1 mL Tris-base (2%, *w*/*v*) and insoluble material was removed by centrifugation. The extent of hydrolysis was analyzed by measuring the absorption at 590 nm in a microplate reader.

### 2.4. Dextran Production

Prior to dextran production, the hydrolytic activity of recombinant glucansucrases was analyzed by hydrolyzing 35 mM *para*-nitrophenyl-α-glucoside in 50 mM sodium acetate buffer (supplemented with 1 mM CaCl_2_, pH 5.5). The amount of *para*-nitrophenol formed was continuously measured in a microplate reader (Infinite M200 PRO, Tecan, Männedorf, Switzerland) at 400 nm. Subsequently, dextrans were produced by adding 1 mU (1 U = 1 μmol of released *para*-nitrophenol/min) dextransucrase to a 500 mM sucrose solution (50 mM sodium acetate buffer, 1 mM CaCl_2_, 0.05% ProClin 300, pH 5.5). After 16 h of incubation, dextrans were precipitated by adding two volumes of ethanol (96% *v*/*v*). Subsequently, the precipitated polysaccharides were recovered by centrifugation and redissolved/resuspended in water. To remove low molecular weight compounds, the solutions/suspensions were dialyzed for 24 h against water (molecular weight cut-off: 3.5 kDa), and freeze-dried.

### 2.5. Dextranase-Mediated Hydrolysis

For dextran hydrolysis, the partially insoluble *L. animalis* and *L. curvatus* dextrans were dissolved in 90:10 DMSO/water (*v*/*v*, 10 mg/mL) and diluted with water (final concentration 1 mg/mL). *L. reuteri* dextrans were completely soluble and thus dissolved in water (1 mg/mL). Hydrolysis was carried out by adding different activities (determined by the above described AZCL dextran assay) to the dextran solutions, followed by 24 h of incubation at 30 °C (SSAL_1145) or 40 °C (all other dextranases). After inactivation of the enzymes by heating to 95 °C for 5 min, the solutions were diluted and analyzed by HPAEC-PAD as described below.

To isolate isomaltotriose and isomaltotetraose for incubation with *Penicillium* sp. dextranase, *L. animalis* dextrans were hydrolyzed with *Penicillium* sp. dextranase (for isomaltotetraose) and BT_3087 (for isomaltotriose) in preparative scale. After inactivation of the enzymes and freeze-drying, the resulting oligosaccharides were separated on a Bio-Gel P-2 column (85 × 2.6 cm) with water as eluent at 1 mL/min and 40 °C. RI-detection was used, fractions collected at fixed time intervals were pooled according to the chromatogram, and freeze-dried. For dextranase hydrolysis, isomaltotriose and isomaltotetraose were dissolved in water (final concentration: 100 µg/mL) and incubated with 0.1 U, 5 U, 25 U, and 100 U as described above.

### 2.6. High Performance Anion Exchange Chromatography

HPAEC-PAD analysis of dextranase hydrolysates was carried out on an ICS-5000 system (Thermo Scientific Dionex, Sunnyvale, CA, USA). A CarboPac PA200 column (250 mm × 3 mm i.d., 5.5 μm particle size, Thermo Scientific Dionex) with water (A), 0.1 M sodium hydroxide (B), 0.1 M sodium hydroxide + 0.5 M sodium acetate (C) as eluents was used for separation. The column was held at 25 °C and the flow rate was 0.4 mL/min. Before every run, the column was washed with 100% C for 10 min and equilibrated with 90% A and 10% B for 20 min. After injection, the following gradient was applied: 0–10 min, isocratic 90% A and 10% B; 10–20 min, linear from 90% A and 10% B to 100% B; 20–45 min, linear from 100% B to 80% B and 20% C; 45–55 min, linear to 100% C; 55–60 min, isocratic 100% C. Previously characterized oligosaccharides [11,12] were used as standard compounds.

## 3. Results and Discussion

### 3.1. Dextranase Production and Characterization

**Figure 1:**
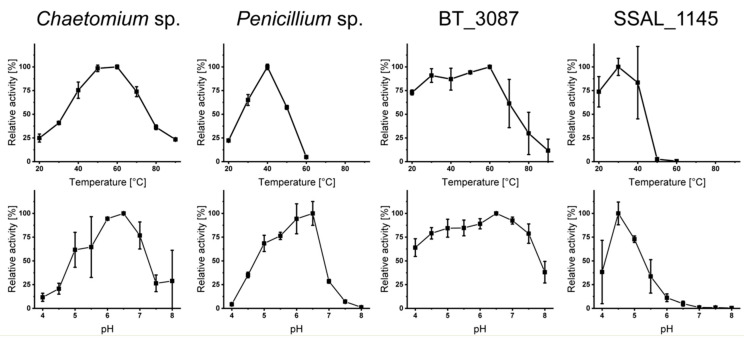
Relative activity of bacterial (SSAL_1145 and BT_3087) and fungal (*Penicillium* sp. and *Chaetomium* sp.) dextranases at different pH values and temperatures, determined photometrically by using AZCL-dextran. The activity at each condition was analyzed in at least two independent experiments.

Heterologous expression of the bacterial dextranases SSAL_1145 and BT_3087 followed by purification with immobilized metal affinity chromatography yielded sufficient amounts of pure enzyme (confirmed by SDS-PAGE, Figure S1). As described above, the focus of this study was on investigating the released oligosaccharides by HPAEC-PAD, however, improper incubation conditions may result in a lack of hydrolysis. Therefore, an assay based on AZCL-dextran was used to determine suitable incubation conditions for the purified and purchased enzymes (Figure 1).

The activity of the enzymes at different temperatures was clearly different. Dextranase from *Chaetomium* sp. showed significant activity between 20 and 90 °C with an optimum between 50–60 °C, which is in good agreement with data from the literature and from the supplier [15,18,19]. In contrast, *Penicillium* sp. dextranase showed a rather narrow activity range between 20 and 60 °C with a maximum around 40 °C. Comparable data were also obtained in previous studies on *Penicillium* sp. dextranases [14,24]. Just as previously described for dextranases from streptococci [26,27,28], SSAL_1145 had its optimum temperature between 25 and 40 °C and was not active at higher temperatures. Notably, dextranase from *B. thetaiotaomicron* showed nearly maximum activity up to 60 °C, thus, the enzyme is very active above the optimum growth conditions of its organism of origin.

Fungal dextranases were most active (>50% relative activity) between pH 5 and 7, which is in good agreement with previous studies [14,15,18,19,21,24]. BT_3087 showed a comparably high activity between pH 4.0 and 7.5, which is again notable with regards to the optimum growth conditions of its organism of origin. In contrast, dextranase from *S. salivarius* was most active at weakly acidic pH (4.0–5.5), which is in good agreement with previous results on dextranases from streptococci [26,27,28]. Because all enzymes were active at weakly acidic pH and because buffers may interfere with the chromatographic analyses, incubations of structurally different dextrans were conducted in bidistilled water (pH 5.5–6.0).

### 3.2. Hydrolysis of Linear Dextrans

To investigate to which degree α-1,6-linked glucose units can be cleaved, dextran from *L. animalis* was used. It has been demonstrated in previous studies [30,34] and in our laboratory, that this polysaccharide is completely linear. However, this also results in some solubility issues, which can mostly be overcome by solvation in DMSO and dilution with water. Nevertheless, incomplete solvation may slow down or inhibit enzymatic hydrolysis to some extent, which was also indicated by lower peak intensities compared to other dextrans.

**Figure 2:**
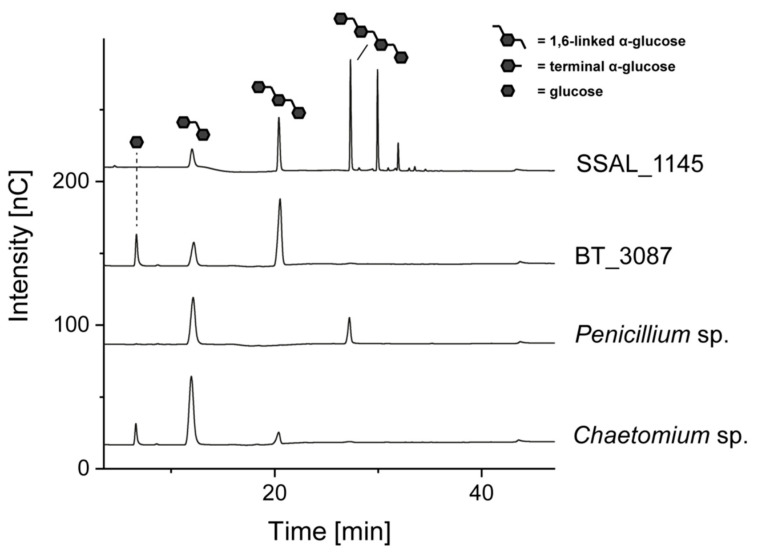
HPAEC-PAD chromatograms of *L. animalis* dextrans after 24 h of incubation with 5 U of dextranases from *Chaetomium* sp., *Penicillium* sp., *B. thetaiotaomicron* (BT_3087), and *S. salivarius* (SSAL_1145). The oligosaccharides shown in the chromatograms were identified by comparison with standard compounds and were not present in the enzyme and dextran solutions. The later eluting peaks in the SSAL_1145 hydrolysate result from linear isomalto-oligosaccharides with a higher degree of polymerization.

In previous studies, we demonstrated that 24 h of incubation at 40 °C and enzyme activity of 5 U dextranase from *Chaetomium* sp./mg dextran is in most cases sufficient for an end point incubation [8,11,12]. Therefore, 5 U and 24 h of incubation at 30 °C (SSAL_1145) and 40 °C (all other dextranases) were initially used for the hydrolysis of *L. animalis* dextran in this study. The HPAEC-PAD chromatograms resulting from these conditions are shown in Figure 2.

**Figure 3:**
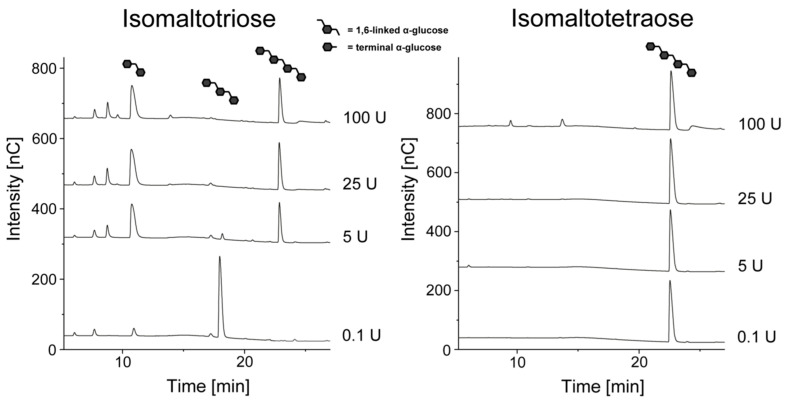
HPAEC-PAD chromatograms of isomaltotriose and isomaltotetraose after 24 h of incubation with different amounts of dextranase from *Penicillium* sp.

As expected, enzymatic hydrolysis with *Chaetomium* sp. dextranase resulted in glucose and isomaltose. Although small amounts of isomaltotriose were detected (probably derived from a delayed hydrolysis of only partially dissolved dextran), this oligosaccharide disappeared with higher enzyme amounts (as already described previously [8,11,12,15]). Hydrolysis with dextranase from *Penicillium* sp. resulted in the formation of isomaltose and isomaltotetraose, whereas glucose or isomaltotriose were not detected. Thus, these results suggest that the *Penicillium* sp. dextranase used is only able to release even-numbered isomalto-oligosaccharides. However, isomaltotriose was detected in some preliminary experiments, therefore, isomaltotriose and isomaltotetraose were purified and separately incubated with different amounts of *Penicillium* sp. dextranase (Figure 3).

Interestingly, isomaltotetraose was not further hydrolyzed by *Penicillium* sp. dextranase. In contrast, 5 U or more of the enzyme converted isomaltotriose to isomaltose and isomaltotetraose, which demonstrates that the enzyme catalyzes a disproportionation reaction with even-numbered isomalto-oligosaccharides as end products. In previous investigations on *Penicillium* sp. dextranases, glucose, isomaltose, isomaltotriose, and isomaltotetraose were described as the main hydrolysis products [14,20,22,23]. The absence of glucose was only described for a dextranase from *Penicillium aculeatum*, but this enzyme yielded isomaltose and isomaltotriose as end products [24]. A condensation reaction was described for a bacterial dextranase from *Arthrobacter globiformis* [35], while it was only hypothesized for a *Penicillium lilalicum* dextranase [22]. However, the latter enzyme was able to cleave isomaltotriose to glucose and isomaltose. Therefore, the preferred production of even-numbered isomalto-oligosaccharides and the catalysis of a disproportionation reaction is a novel property of *Penicillium* sp. dextranases.

**Figure 4:**
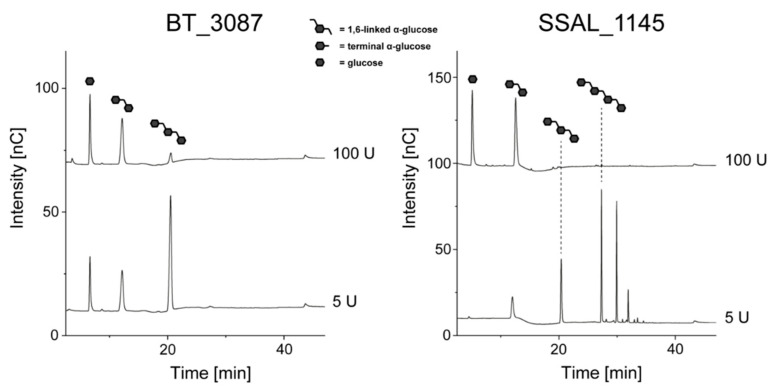
HPAEC-PAD chromatograms of *L. animalis* dextrans after 24 h of incubation with 5 U and 100 U of dextranases from *B. thetaiotaomicron* (BT_3087) and *S. salivarius* (SSAL_1145). The oligosaccharides shown in the chromatograms were identified by comparison with standard compounds and were not present in the enzyme and dextran solutions. The later eluting peaks in the SSAL_1145 hydrolysates result from linear isomalto-oligosaccharides with a higher degree of polymerization.

Because the HPAEC-PAD chromatograms obtained from the hydrolysis of *L. animalis* dextran with BT_3087 and SSAL_1145 contained isomaltotriose as well as higher oligosaccharides (in case of SSAL_1145), a higher enzyme amount (100 U) was applied to investigate if further hydrolysis is possible (Figure 4).

As it can be seen from the chromatograms, higher activities of both BT_3087 and SSAL_1145 resulted in (almost) complete degradation of higher oligosaccharides and isomaltotriose to isomaltose and glucose. Therefore, both enzymes eventually yield the same end products as dextranase from *Chaetomium* sp. However, different products may be obtained after the hydrolysis of branched dextrans.

### 3.3. Hydrolysis of O3- and O4-Branched Dextrans

**Figure 5:**
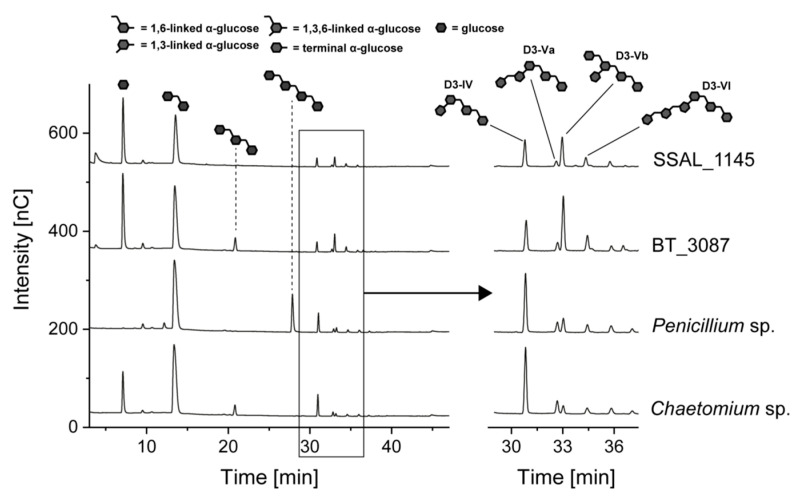
HPAEC-PAD chromatograms of *L. curvatus* dextrans after 24 h of incubation with 5 U of dextranases from *Chaetomium* sp., *Penicillium* sp. and 100 U of dextranases from *B. thetaiotaomicron* (BT_3087), and *S. salivarius* (SSAL_1145). The oligosaccharides shown in the chromatograms were identified by comparison with previously characterized standard compounds [11] and were not present in the enzyme and dextran solutions.

The enzymatic hydrolysis of *O*3-branched dextrans was investigated by using dextrans produced by glucansucrase from *L. curvatus* TMW 1.624. Oligosaccharides that result from the hydrolysis of these dextrans with *Chaetomium* sp. dextranase were previously isolated and characterized in detail [11]. To achieve an end-point hydrolysis of the oligo- and polysaccharides, the enzyme amounts needed for complete hydrolysis of *L. animalis* dextrans were used (bacterial dextranases: 100 U, fungal dextranases: 5 U, Figure 5).

Dextranase from *Chaetomium* sp. yielded the expected oligosaccharide pattern (except for the minor amounts of isomaltotriose). With regards to the branched oligosaccharides, *Penicillium* sp. dextranase yielded a similar product pattern and only slightly varying peak intensities were observed. In addition, this enzyme only liberated isomaltose and isomaltotetraose, which is in good agreement with the results described above. As expected, the bacterial dextranases yielded glucose and isomaltose as the main products, however, clearly different intensities were detected for the branched oligosaccharides compared to the dextranases from *Chaetomium* sp. and *Penicillium* sp. While D3-Va and D3-VI were of comparable abundance, D3-IV showed a lower peak intensity and D3-Vb showed a clearly higher peak intensity compared to the hydrolysates obtained with fungal dextranases. Both oligosaccharides represent monomeric, *O*3-bound side chains and only differ in the attachment of an additional non-reducing glucose unit bound to the *O*3-branched backbone unit. Therefore, it is likely that the two oligosaccharides represent the same portion of monomeric side chains, but different modes of actions of the enzymes yield varying abundances of the corresponding oligosaccharides. This could be the result from the attack of different sites within the α-1,6-linked areas of the dextrans, which would lead to different intermediate products and thus in an increased formation of D3-Vb, which cannot be hydrolyzed any further. However, the oligosaccharide profiles obtained with bacterial dextranases also clearly indicate the predominant abundance of monomeric side chains.

**Figure 6:**
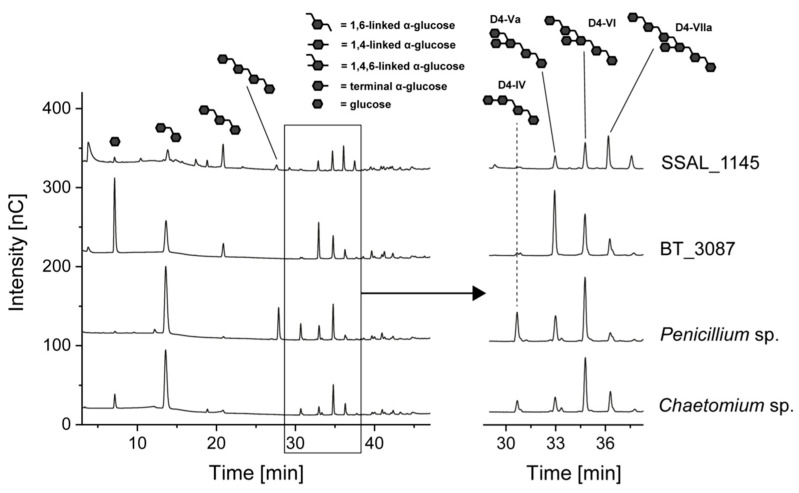
HPAEC-PAD chromatograms of *L. reuteri* dextrans after 24 h of incubation with 5 U of dextranases from *Chaetomium* sp., *Penicillium* sp. and 100 U of dextranases from *B. thetaiotaomicron* (BT_3087), and *S. salivarius* (SSAL_1145). The oligosaccharides shown in the chromatograms were identified by comparison with previously characterized standard compounds [11,12] and were not present in the enzyme and dextran solutions.

The *O*4-branched dextrans formed by glucansucrase from *L. reuteri* TMW 1.106 were also investigated because they represent a rather rare dextran type and because several of the enzymatically released oligosaccharides were previously characterized [11,12]. The resulting HPAEC-PAD chromatograms are shown in Figure 6.

**Figure 7:**
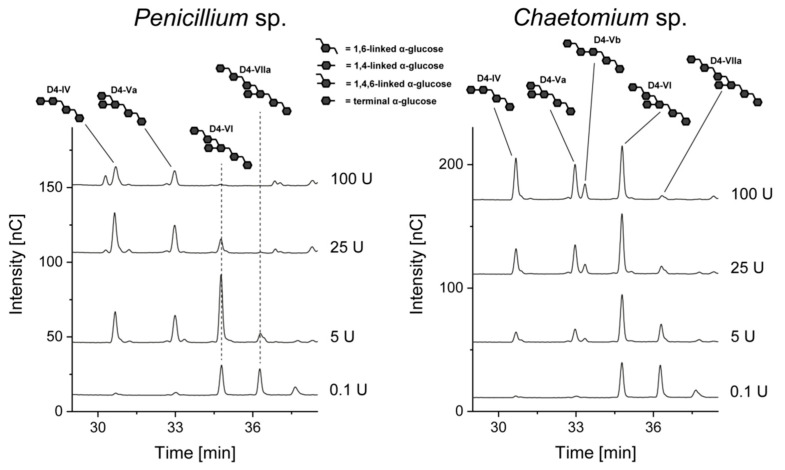
HPAEC-PAD chromatograms of *L. reuteri* dextrans after 24 h of incubation with different amounts of dextranase from *Chaetomium* sp. and *Penicillium* sp. The oligosaccharides shown in the chromatograms were identified by comparison with previously characterized standard compounds [11,12] and were not present in the enzyme and dextran solutions.

The chromatogram obtained from the hydrolysis of *L. reuteri* dextrans with 5 U of *Chaetomium* sp. dextranase yielded the previously described product pattern [11]. A comparable pattern was detected in the hydrolysate obtained with *Penicillium* sp. dextranase, however, D4-IV and D4-Va had a higher intensity, whereas the other oligomers showed a lower intensity. Because the hexa- and heptameric oligosaccharides contain two or three 1,6-linked glucose units on the non-reducing end, the higher abundance of D4-IV and D4-Va may result from an elevated and possibly ongoing hydrolysis of high molecular weight compounds. To evaluate this, *L. reuteri* dextrans were incubated with a higher enzyme amount (100 U) of *Penicillium* sp. dextranase and, as a comparison, dextranase from *Chaetomium* sp. (Figure 7).

In case of *Penicillium* sp. dextranase, an activity dependent degradation of higher oligosaccharides can be observed. While D4-VI and D4-VIIa are the clearly predominating products at low enzyme activities, they are completely degraded to D4-IV and D4-Va at higher activities. Therefore, isomaltose units are liberated from the non-reducing ends of the higher oligosaccharides. Interestingly, higher activities of *Chaetomium* sp. dextranase also led to some further hydrolysis of D4-VIIa (although linear oligosaccharides are usually completely hydrolyzed at this activity level). As it can be seen in the chromatograms, this compound (and most likely other later eluting branched oligosaccharides) were degraded to D4-IV and D4-Va. However, the activity on D4-VIIa seems to be rather low, as only a slight improvement is observed from 5 U over 25 U to 100 U. Nonetheless, *Chaetomium* sp. dextranase seems to be able to remove isomaltosyl or isomaltotriosyl units from the non-reducing end of branched oligosaccharides. Notably, the portion of D4-Vb also increased with increasing enzyme activity. This is most likely a result of the hydrolysis of higher oligosaccharides with dimeric side chains (such as D4-VIIb, which may be present in trace amounts). Therefore, information on dimeric, 1,6-linked side chains can also be derived from the hydrolysates obtained with higher enzyme activities.

The two bacterial dextranases showed clearly different product patterns compared to dextranases from *Chaetomium* sp. and *Penicillium* sp. Notably, D4-IV and D4-Vb (no terminal, *O*6-bound glucose unit) were not detected in significant amounts in the hydrolysates obtained with both enzymes; thus, they are (unlike the fungal dextranases) not able to cleave the 1,6-linkage in a 1,4,6-linked glucose unit. The main products obtained from the hydrolysis with BT_3087 were D4-Va, D4-VI, and D4-VIIa and the peak intensity decreased from D4-Va to D4-VIIa. SSAL_1145 also yielded these oligosaccharides, but the peak intensity decreased from D4-VIIa to D4-Va. In addition, an unknown oligosaccharide which eluted shortly after D4-VIIa was detected. Therefore, SSAL_1145 showed a clearly lower activity on the *O*4-branched dextrans than the other enzymes, which is also indicated by the generally low peak intensities and the low abundance of linear isomalto-oligosaccharides.

## 4. Conclusions

Overall, the results of this study demonstrated the diversity of dextranases from different origins. With regards to the incubation conditions, the high activity of all enzymes around 40 °C and the weakly acidic pH is in good agreement with previous studies and in most cases the preferred growth conditions of the organisms of origin. Hydrolysis of linear dextrans showed that dextranases from *B. thetaiotaomicron*, *S. salivarius*, and *Chaetomium* sp. eventually lead to the formation of isomaltose. However, isomaltose and isomaltotetraose were detected as the end products of the hydrolysis with *Penicillium* sp. dextranase. This unique product spectrum is the result of a disproportionation reaction, in which isomaltotriose is converted to isomaltose and isomaltotetraose. For *O*3-branched dextrans, the product patterns obtained with fungal and bacterial dextranases were comparable within the two groups but different between them. Namely, dextranases from *Chaetomium* sp. and *Penicillium* sp. were able to hydrolyze *O*3-branched dextrans to a higher extent. However, the same conclusions on the dextran structures could be drawn from the oligosaccharide profiles. In addition, bacterial dextranases also hydrolyzed *O*4-branched dextrans to a minor extent compared to fungal dextranases, which resulted in higher portions of longer branched oligosaccharides. Notably, *Penicillium* sp. dextranase was able to hydrolyze this dextran type to a clearly higher degree than *Chaetomium* sp. dextranase. Therefore, the enzyme applied for structural analysis or technological purposes has be to chosen carefully. However, the differences between the enzymes can also be used to selectively produce certain oligosaccharides in higher yields.

## Supplementary Materials

The following are available online at https://www.mdpi.com/2304-8158/10/2/244/s1, Figure S1: SDS-PAGE analysis of the two recombinant bacterial dextranases SSAL_1145 and BT_3087.

## References

[1] Naessens M., Cerdobbel A., Soetaert W., Vandamme E. (2005). *Leuconostoc* dextransucrase and dextran: Production, properties and applications. J. Chem. Technol. Biotechnol..

[2] Leemhuis H., Pijning T., Dobruchowska J.M., Van Leeuwen S.S., Kralj S., Dijkstra B.W., Dijkhuizen L. (2013). Glucansucrases: Three-dimensional structures, reactions, mechanism, α-glucan analysis and their implications in biotechnology and food applications. J. Biotechnol..

[3] Torino M.I., Font G., Mozzi F. (2015). Biopolymers from lactic acid bacteria. Novel applications in foods and beverages. Front. Microbiol..

[4] Zannini E., Waters D.M., Coffey A., Arendt E.K. (2016). Production, properties, and industrial food application of lactic acid bacteria-derived exopolysaccharides. Appl. Microbiol. Biotechnol..

[5] Khalikova E., Susi P., Korpela T. (2005). Microbial Dextran-Hydrolyzing Enzymes: Fundamentals and Applications. Microbiol. Mol. Biol. Rev..

[6] Katina K., Maina N.H., Juvonen R., Flander L., Johansson L., Virkki L., Tenkanen M., Laitila A. (2009). In situ production and analysis of *Weissella confusa* dextran in wheat sourdough. Food Microbiol..

[7] Maina N.H., Virkki L., Pynnönen H., Maaheimo H., Tenkanen M. (2011). Structural Analysis of Enzyme-Resistant Isomaltooligosaccharides Reveals the Elongation of α-(1→3)-Linked Branches in *Weissella confusa* Dextran. Biomacromolecules.

[8] Fels L., Jakob F., Vogel R.F., Wefers D. (2018). Structural characterization of the exopolysaccharides from water kefir. Carbohydr. Polym..

[9] Xu D., Fels L., Wefers D., Behr J., Jakob F., Vogel R.F. (2018). *Lactobacillus hordei* dextrans induce *Saccharomyces cerevisiae* aggregation and network formation on hydrophilic surfaces. Int. J. Biol. Macromol..

[10] Bechtner J., Wefers D., Schmid J., Vogel R.F., Jakob F. (2019). Identification and comparison of two closely related dextransucrases released by water kefir borne *Lactobacillus hordei* TMW 1.1822 and *Lactobacillus nagelii* TMW 1.1827. Microbiology.

[11] Münkel F., Wefers D. (2019). Fine structures of different dextrans assessed by isolation and characterization of endo-dextranase liberated isomalto-oligosaccharides. Carbohydr. Polym..

[12] Münkel F., Bechtner J., Eckel V., Fischer A., Herbi F., Jakob F., Wefers D. (2019). Detailed Structural Characterization of Glucans Produced by Glucansucrases from *Leuconostoc citreum* TMW 2.1194. J. Agric. Food Chem..

[13] Münkel F., Fischer A., Wefers D. (2020). Structural characterization of mixed-linkage α-glucans produced by mutants of *Lactobacillus reuteri* TMW 1.106 dextransucrase. Carbohydr. Polym..

[14] Das D.K., Dutta S.K. (1996). Purification, biochemical characterisation and mode of action of an extracellular endo-dextranase from the culture filtrate of *Penicillium lilacinum*. Int. J. Biochem. Cell Biol..

[15] Hattori A., Ishibashi K., Minato S. (1981). The purification and characterization of the dextranase of *Chaetomium gracile*. Agric. Biol. Chem..

[16] Larsson A.M., Andersson R., Stahlberg J., Kenne L., Jones T.A. (2003). Dextranase from *Penicillum minioluteum*: Reaction course, crystal structure, and product complex. Structure.

[17] Sugiura M., Ito A., Ogiso T., Kato K., Asano H. (1973). Studies on dextranase—Purification of dextranase from *Penicillium funiculosum* and its enzymatic properties. Biochim. Biophys. Acta.

[18] Virgen-Ortíz J., Ibarra-Junquera V., Escalanteminakata P., Ornelas-Paz J.D.J., Osunacastro J.A., González-Potes A. (2015). Kinetics and thermodynamic of the purified dextranase from *Chaetomium erraticum*. J. Mol. Catal. B Enzym..

[19] Yang L., Zhou N., Tian Y. (2018). Purification, characterization, and biocatalytic potential of a novel dextranase from *Chaetomium globosum*. Biotechnol. Lett..

[20] Yilan O., Sun X., Du K., Ouyang Y., Wu C., Xu N., Linhardt R.J., Zhang Z. (2015). UP-HILIC-MS/MS to Determine the Action Pattern of *Penicillium* sp. Dextranase. J. Am. Soc. Mass Spectrom..

[21] Erhardt F.A., Stammen S., Jördening H.-J. (2008). Production, characterization and (co-)immobilization of dextranase from *Penicillium aculeatum*. Biotechnol. Lett..

[22] Walker G.J., Dewar M.D. (1975). The action pattern of *Penicillium lilacinum* dextranase. Carbohydr. Res..

[23] Hiraoka N., Tsuji H., Fukumoto J., Yamamoto T., Tsuru D. (2009). Studies on Mold Dextranases: Some Physicochemical Properties and Substrate Specificity of Dextranases Obtained from *Aspergillus carneus* and *Penicillium luteum*. Int. J. Pept. Protein Res..

[24] Prabhu M., Prabhu K.A. (1984). Studies on dextranase from *Penicillium aculeatum*. Enzyme Microb. Technol..

[25] Kim Y.-M., Yamamoto E., Kang M.-S., Nakai H., Saburi W., Okuyama M., Mori H., Funane K., Momma M., Fujimoto Z. (2012). *Bacteroides thetaiotaomicron* VPI-5482 glycoside hydrolase family 66 homolog catalyzes dextranolytic and cyclization reactions. FEBS J..

[26] Igarashi T., Morisaki H., Goto N. (2004). Molecular Characterization of Dextranase from *Streptococcus rattus*. Microbiol. Immunol..

[27] Wanda S.Y., Curtiss R. (1994). Purification and characterization of *Streptococcus sobrinus* dextranase produced in recombinant Escherichia coli and sequence analysis of the dextranase gene. J. Bacteriol..

[28] Pulkownik A., Walker G.J. (1977). Purification and substrate specificity of an endo-dextranase of *Streptococcus mutans* K1-R. Carbohydr. Res..

[29] Van Bueren A.L., Saraf A., Martens E.C., Dijkhuizen L. (2015). Differential Metabolism of Exopolysaccharides from Probiotic Lactobacilli by the Human Gut Symbiont *Bacteroides thetaiotaomicron*. Appl. Environ. Microbiol..

[30] Rühmkorf C., Rübsam H., Becker T., Bork C., Voiges K., Mischnick P., Brandt M.J., Vogel R.F. (2012). Effect of structurally different microbial homoexopolysaccharides on the quality of gluten-free bread. Eur. Food Res. Technol..

[31] Aslanidis C., De Jong P.J. (1990). Ligation-independent cloning of PCR products (LIC-PCR). Nucleic Acids Res..

[32] Burgess-Brown N.A., Sharma S., Sobott F., Loenarz C., Oppermann U., Gileadi O. (2008). Codon optimization can improve expression of human genes in *Escherichia coli*: A multi-gene study. Protein Expr. Purif..

[33] Stols L., Gu M.Y., Dieckman L., Raffen R., Collart F.R., Donnelly M.I. (2002). A new vector for high-throughput, ligation-independent cloning encoding a tobacco etch virus protease cleavage site. Protein Expr. Purif..

[34] Rühmkorf C., Bork C., Mischnick P., Rübsam H., Becker T., Vogel R.F. (2013). Identification of *Lactobacillus curvatus* TMW 1.624 dextransucrase and comparative characterization with *Lactobacillus reuteri* TMW 1.106 and *Lactobacillus animalis* TMW 1.971 dextransucrases. Food Microbiol..

[35] Sawai T., Niwa Y. (1975). Transisomaltosylation activity of a bacterial isomalto-dextranase. Agric. Biol. Chem..

