# Supplementary figures and images for: Products Released from Structurally Different Dextrans by Bacterial and Fungal Dextranases
Source: Foods. 2021 Jan 26;10(2):244. doi: 10.3390/foods10020244 (PMC7911647; doi:10.3390/foods10020244)

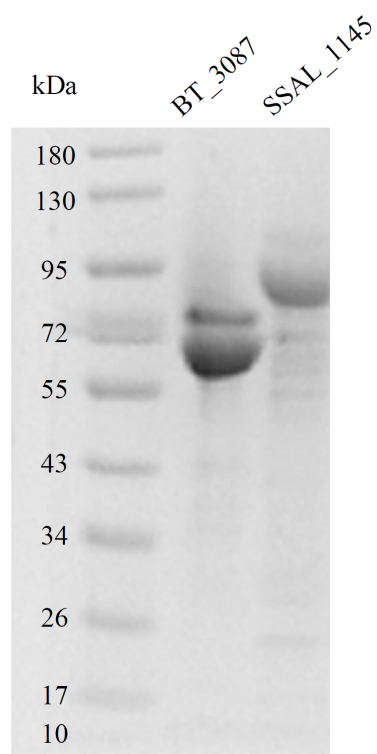

**Figure S1.** SDS-PAGE analysis of the two recombinant bacterial dextranases SSAL\_1145 and BT\_3087.

Supplement: Supplementary file 1 [file foods-10-00244-s001.pdf]
